# Intravenous Dexmedetomidine Provides Superior Patient Comfort and Tolerance Compared to Intravenous Midazolam in Patients Undergoing Flexible Bronchoscopy

**DOI:** 10.1155/2015/727530

**Published:** 2015-10-12

**Authors:** Umesh Goneppanavar, Rahul Magazine, Bhavya Periyadka Janardhana, Shreepathi Krishna Achar

**Affiliations:** ^1^Department of Anaesthesiology, Dharwad Institute of Mental Health and Neurosciences, Dharwad, Karnataka 580008, India; ^2^Department of Pulmonary Medicine, Kasturba Medical College, Manipal University, Manipal, Karnataka 576104, India; ^3^Department of Anaesthesiology, Kasturba Medical College, Manipal University, Manipal, Karnataka 576104, India

## Abstract

Dexmedetomidine, an *α*_2_ agonist, has demonstrated its effectiveness as a sedative during awake intubation, but its utility in fiberoptic bronchoscopy (FOB) is not clear. We evaluated the effects of midazolam and dexmedetomidine on patient's response to FOB. The patients received either midazolam, 0.02 mg/kg (group M, *n* = 27), or dexmedetomidine, 1 *µ*g/kg (group D, *n* = 27). A composite score of five different parameters and a numerical rating scale (NRS) for pain intensity and distress were used to assess patient response during FOB. Patients rated the quality of sedation and level of discomfort 24 h after the procedure. Ease of bronchoscopy, rescue medication requirement, and haemodynamic variables were noted. Ideal or acceptable composite score was observed in 15 and 26 patients, respectively, in group M (14.48 ± 3.65) and group D (9.41 ± 3.13), *p* < 0.001. NRS showed that 11 patients in group M had severe pain and discomfort as compared to one patient with severe pain and two with severe discomfort in group D during the procedure, *p* < 0.001. Rescue midazolam requirement was significantly higher in group M (*p* = 0.023). We conclude that during FOB, under topical airway anaesthesia, IV dexmedetomidine (1 *µ*g/kg) provides superior patient comfort and tolerance as compared to IV midazolam (0.02 mg/kg).

## 1. Introduction

Flexible fiberoptic bronchoscope has replaced rigid bronchoscopy as the technique of choice for evaluation of the airway. The reasons are reduced requirement of sedation, lesser complications related to the procedure, and better acceptability by the patients [1]. However, patients can still experience variable degree of pain, discomfort, and phobia, resulting in difficulty in performing the procedure and reducing its diagnostic utility [2]. Topical anaesthesia of airway alone is, often, insufficient to provide desirable patient comfort [3]. Therefore, in addition, appropriate sedation to enhance patient comfort and satisfaction is required [3–6]. Moreover, flexible bronchoscopy can cause sympathetic stimulation resulting in tachycardia, hypertension, arrhythmias, and increased oxygen demand that can be deleterious to an already diseased heart [7]. Hence, current guidelines on flexible bronchoscopy recommend offering sedation to all patients [8].

Since the duration of the procedure is short and is performed mostly on outpatient basis, the ideal sedative should have rapid onset, short duration, rapid offset, and no adverse effects, but ensuring sedation sufficient to blunt the sympathetic responses and airway irritability. A variety of pharmacological agents including benzodiazepines, opioids, sympatholytic drugs, and propofol, alone or in combination, have been found to be useful. However, several complications are also attributed to their use, and, hence, the search is still on for a better sedative [9–14]. British Thoracic Society Guidelines have suggested the use of intravenous midazolam as 2 mg bolus prior to the procedure [8]. Midazolam is one of the most widely used drugs for sedation during bronchoscopy in view of its amnesic and anxiolytic properties along with its favourable pharmacokinetic profile such as rapid-onset and short-lasting depressant action on the central nervous system. Moreover, it has the advantage that its effect can be rapidly counteracted using flumazenil, a competitive antagonist for benzodiazepine receptors in situations of overdose. Recent evidence shows that dexmedetomidine, an *α*_2_ agonist, can provide excellent sedation (anxiolysis, sedation, and analgesia without the risk of respiratory depression) during awake fiberoptic intubation in the management of difficult airway and also to patients in critical care units [15–19]. Dexmedetomidine has been evaluated in a few studies for its sedative effects during flexible fiberoptic bronchoscopy and has been found to be useful in enhancing patient comfort without the risk of respiratory depression [14, 20].

This study evaluated the effectiveness of midazolam or dexmedetomidine in improving patient comfort during flexible fiberoptic bronchoscopy.

## 2. Aims and Objective

The objective is to evaluate the effects of two intravenously administered pharmacological interventions, that is, midazolam and dexmedetomidine, on patient response to flexible bronchoscopy. Main outcome measure was a composite score of five different parameters on which patient response was assessed with the goal of identifying the drug that could provide better patient tolerance and improved fibrescopist comfort without compromising patient safety.

## 3. Materials and Methods

This study was prospective, randomized, and double blinded. Approval was obtained from the institutional ethics committee of our hospital. It was conducted in 54 patients who were considered appropriate for the study methodology. Written informed consent was obtained from the patients who were willing to participate in the study, after they had read and understood the patient information sheet. All patients in the age group 18 to 70 years requiring flexible bronchoscopic evaluation of the airway in the Department of Pulmonary Medicine of our institution were considered for the study. Those who were excluded from participating in the study included the ones with known or suspected allergy to any of the study drugs (dexmedetomidine, midazolam) and those with seizure disorder, moderate to severe chronic obstructive pulmonary disease, disease condition that would confuse the behaviour assessment, renal (with serum creatinine > 2 mg/dL) or hepatic impairment (elevated liver enzymes > 2 times normal) and haemodynamic instability (bradycardia with HR < 50 bpm or hypotension with SBP < 90 mmHg), or seriously ill patients with American Society of Anesthesiologists' physical status 4.

The patients were randomized into two groups, midazolam (0.02 mg/kg) and dexmedetomidine (1 *µ*g/kg), as per a computer generated randomization table. Two drops of oxymetazoline (0.05%) were administered into each nostril half an hour prior to beginning of the study. All patients were monitored: pulse rate, noninvasive blood pressure (NIBP), electrocardiogram, oxygen saturation with pulse oximeter, respiratory rate and sedation status using the Ramsay Sedation Score (RSS). After obtaining baseline values for these parameters, an intravenous access was established in the upper limb. The study drugs were loaded in one of the two syringes labelled A and B to help in blinding (*syringe A* had a volume of 10 mL and contained either dexmedetomidine 1 *µ*g/kg diluted in normal saline or only normal saline;* syringe B* had a volume of 2 mL and contained either midazolam 0.02 mg/kg diluted with normal saline or normal saline). The study was begun with infusion of contents of syringe A over 10 minutes. At the beginning of the 9th minute, contents of syringe B were administered as a bolus. At the end of the 10th minute, bronchoscopy was commenced. Two mL lignocaine jelly (2%) was applied to the more patent nostril following which the fibrescope (FFB) was passed through the nostril. The throat and vocal cords were sprayed with 2 mL aliquots of 2% lignocaine solution (total 4 mL). After waiting for a minute, the fibrescope was advanced below the vocal cords where 1 mL, 2% lignocaine was sprayed. The fibrescope was advanced till carina where 1 mL, 2% lignocaine was sprayed into each main bronchus (total 2 mL). Once the patient coughing subsided, the fibrescope was advanced into either bronchi till tertiary segments were evaluated and the procedure for which the patient was scheduled was completed. In the event of patient having an RSS 1 after the topicalisation of the airway was complete, intravenous midazolam 1 mg bolus was administered (as rescue medication), which was kept in an open label syringe. At least two-minute gap between two doses of midazolam was maintained and the patient's level of sedation was constantly assessed and maintained between RSS 2 and 3.

The study involved three different observers.* Observer 1* who picked up the randomization slip prepared the study drug, accordingly ensuring blinding (labelled the syringes A and B). Observer 1 did not have any further involvement with the study subject.* Observer 2* performed the bronchoscopy and* observer 3* assessed the patients, explained study methodology, obtained consent, administered the study drugs, and recorded the study parameters. Both observers 2 and 3 were blinded to the study drug and they were constant throughout the study.

Patient response to bronchoscopy was assessed using five different parameters which together contributed to the composite score (Table 1). For clinical and statistical purposes, the composite score was considered ideal when the score was 5–10, acceptable when the score was 11–15, and unacceptable when the score was >15. This was used as our primary outcome measure for the study. Patient response to bronchoscopy was also assessed using a numerical rating scale (for pain intensity and distress) prior to, during, and 10 min after bronchoscopy. A score of 0 on the NRS meant that the patient was comfortable and cooperative while a score of 10 was considered the worst patient response. Numerical rating scale for pain intensity and distress (assessed during procedure by observer 3 and patient response obtained at 10 min following the procedure) is further graded for statistical analysis as mild when the score was between 0 and 3, moderate when the score was between 4 and 7, and severe when the score was between 8 and 10. Sedation state was assessed using Ramsay Sedation Score prior to, during, and 10 min after procedure. Observer 2 who performed flexible bronchoscopy on all the study subjects assessed the ease of bronchoscopy as easy, slightly difficult, or very difficult. Approximately 24 h after the procedure, all patients were asked to give their opinion regarding the quality of sedation (excellent/good/fair/poor) and level of discomfort during the procedure (none to mild, moderate, and severe). The duration of bronchoscopy, number of doses of rescue medication required, haemodynamic variables such as heart rate (HR), systolic blood pressure (SBP), diastolic blood pressure (DBP), and any change in rhythm pattern during the study period were noted. Starting just prior to bronchoscopy, the haemodynamic variables were noted every 2 minutes till the end of bronchoscopy and 10 minutes after the procedure. Patients were also monitored for any oxygen desaturation (SpO_2_ < 93%) or hypoventilation (RR < 8/min).

### 3.1. Sample Size Estimation

A pilot study was done that included four patients in midazolam group and three patients in dexmedetomidine group. The results of pilot study showed a composite score difference of 2 between the groups. Based on this data, it was estimated that 26 patients were needed in each group to have a power of 80% at 95% confidence interval for a composite score difference of 3.

### 3.2. Statistical Analysis

After collecting the data, mean and standard deviation were calculated as appropriate. Continuous variables like age, height, weight, BMI, and duration of bronchoscopy between groups were compared using independent *t*-test. Whereas categorical data like gender, numerical rating scale, Ramsay Sedation Score, requirement of rescue medication, ease of bronchoscopy, and postprocedure patient assessment of bronchoscopy were compared between groups using either Fisher's exact test or Chi-square test, composite score was analysed using Mann-Whitney *U* test. All the analyses were performed on SPSS version 18.0 (SPSS Inc., Chicago, IL, USA). A *p* value of <0.05 was considered statistically significant.

## 4. Results

A total of 54 patients (27 in each group) participated and all of them completed the study. The age, gender, weight, and body mass index between the groups were comparable while patients in group M were taller than those in group D. All patients underwent diagnostic evaluation of the tracheobronchial tree. The mean duration of bronchoscopy was comparable between the two groups with 8.19 ± 2.53 min in group M and 8.93 ± 2.96 in group D (Table 2).

The composite score (a reflector towards patient cooperation and tolerance to the procedure) was ideal or acceptable in 15 patients in group M and 26 patients in group D. The mean scores were 14.48 ± 3.65 in group M and 9.41 ± 3.13 in group D (*p* < 0.001, Table 3). Individual components of the composite score showed that >25% patients in group M had unacceptable scores (score of 4 or 5) in four out of five segments (except sedation) while most patients (>90%) in group D had ideal or acceptable scores in all segments (Table 4).

NRS showed that in group M eleven patients had severe pain and discomfort as compared to one patient with severe pain and two with severe discomfort in group D during the procedure (Table 5, *p* value < 0.001, Chi-square test; statistically significant). None had pain or discomfort before the beginning of the procedure in either group. One patient had moderate pain and eight patients had moderate distress 10 min following the procedure in group M while none had severe pain or distress. In contrast, group D had none experiencing moderate or severe pain while one patient had moderate discomfort 10 min following bronchoscopy. The number of patients having persistent distress following bronchoscopy was significantly higher in group M (*p* = 0.024, Table 5).

The bronchoscopy was assessed to be easy in 6, slightly difficult in 11, and very difficult in 10 patients in group M while the bronchoscopy was easy in 15, slightly difficult in 10, and very difficult in 2 patients in group D (Chi-square test, *p* = 0.010; statistically significant, Table 6). Rescue bolus midazolam requirement was significantly higher in group M where eight patients required single bolus, two patients required two boluses, and one patient required three boluses while two patients in group D required a single bolus (*p* value = 0.023, Fisher's exact test; statistically significant, Table 6).

A Ramsay Sedation Score of 2-3 was desired during and after the study. None in group M had deeper level of sedation than necessary at the beginning of the procedure while three patients in group D had a RSS of 4 at the beginning of bronchoscopy. An RSS score of 1 (anxious and agitated or restless or both) was found during the procedure in 11 patients in group M and in two patients in group D. One patient in group M had RSS 4 at 10 min following bronchoscopy (the same patient had received three rescue boluses of midazolam during the procedure) and four patients in group D had RSS 4 at 10 min following bronchoscopy (Table 6). All these patients were observed and monitored till they recovered from deep levels of sedation. None developed any complication.

**Figure 1:**
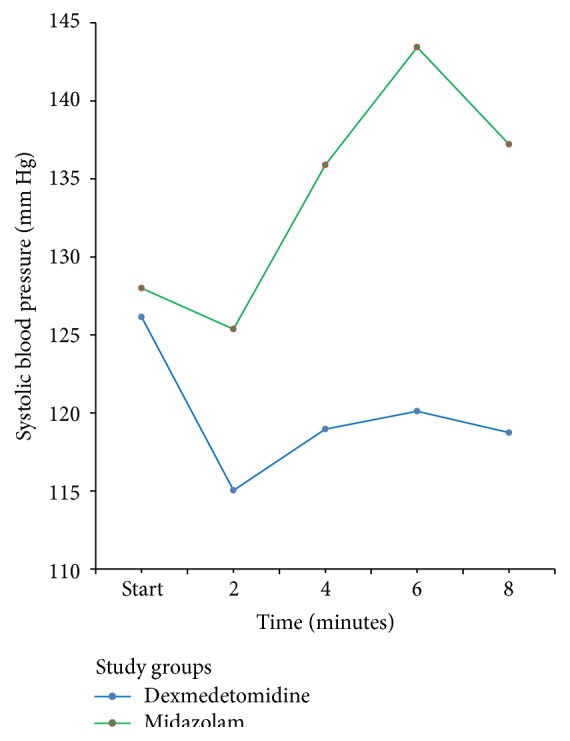
Systolic blood pressure in relation to time.

None of the patients had any episodes of desaturation or significant bradycardia. One patient in group D had hypotension following bronchoscopy that responded to a single bolus of 250 mL crystalloid. When estimated marginal means for systolic blood pressure were compared between the two groups, the SBP was within 20% from baseline in the dexmedetomidine group at all times while there was elevation of SBP to >20% from baseline in the midazolam group at the fourth minute after starting the procedure (Figure 1). Since a significant number of procedures lasted ≤6  min, we could not compare the data beyond this time frame. However, SBP at 10 min following bronchoscopy was comparable between the groups (112 ± 18 mmHg in dexmedetomidine group versus 131 ± 19 mmHg in midazolam group, *p* = 0.657). Postoperative questionnaire of patients revealed that 7 and 21 patients in group M and group D, respectively, felt that the quality of sedation was excellent/good. Others felt that it was either fair or poor. Similarly, when asked about the amount of discomfort the patients perceived during the procedure, 12 and 23 patients in group M and group D, respectively, felt none or mild discomfort. Others felt that they experienced moderate to severe discomfort during the procedure (Table 6).

## 5. Discussion

Flexible bronchoscopy for evaluation of tracheobronchial tree is a very commonly performed procedure by pulmonologists and in many countries it is done on outpatient basis. The protocol of our hospital was to admit the patients overnight following the procedure and this allowed us to evaluate the patients 24 h after the procedure.

In our study, we evaluated the patients using several parameters that included assessment of patient responses during bronchoscopy based on five different parameters (composite score), bronchoscopist's evaluation of the conditions for bronchoscopy, Ramsay Sedation Score, NRS for evaluating pain and distress experienced by the patient during the procedure, and patient's own evaluation of the procedural sedation 24 h following the procedure (once they were completely free of the effects of the sedative agents). Further, haemodynamic response to bronchoscopy was also evaluated. Other studies that evaluated procedural sedation with midazolam or dexmedetomidine during bronchoscopy have considered number of episodes of oxygen desaturation, number of times patient required assistance to maintain the patency of the airway, cough and satisfaction scores by patients and bronchoscopists, level of sedation, number of or type of rescue doses needed, haemodynamic responses, numerical or visual analogue scale score for assessment by patient or observer to rate the comfort or satisfaction, and postoperative patient questionnaire [9–12, 14, 15]. Strength of our study is that it was double blinded and the patient tolerance to bronchoscopy was evaluated mainly using a composite score believing that this would reflect many aspects of patient discomfort to bronchoscopy while recording only one aspect such as cough or patient/operator evaluation of discomfort might appear too subjective and might miss out other manifestations of patient discomfort.

Analysis of various parameters in our study shows that dexmedetomidine provided superior and acceptable patient tolerance and comfort, bronchoscopy conditions, and level of sedation with an episode of hypotension (that responded to single fluid bolus) as the only adverse event while midazolam failed to provide satisfactory patient comfort or bronchoscopy conditions in a large number of patients. Other studies have found better patient acceptance and bronchoscopy conditions with midazolam, but most of them are with much higher doses of midazolam (2–2.5 mg boluses followed by 1 mg supplements or >0.03 mg/kg bolus). However, these studies have also shown that it was achieved with serious adverse effects such as oxygen desaturation [11, 12, 21, 22], inability on part of the patient to maintain the airway requiring verbal/tactile stimulation or airway manoeuvres or ventilator assistance or flumazenil for antagonizing the effects of midazolam [11, 21], considerable reduction in both the inspiratory and expiratory muscle strengths (both maximum inspiratory and expiratory pressures) [23], and hiccups [24]. This can be a very important limiting factor and deterrent for the use of higher doses of midazolam (>0.02 mg/kg or >1-2 mg bolus) when the responsibility for monitoring and managing the adverse effects rests on the pulmonologist or otorhinolaryngologist. Further, BTS Guidelines suggest that a better way to provide sedation is by incremental administration of the drug rather than a single bolus dose. Our ultimate goal is to find a suitable drug at a dose that does not produce any serious adverse effects so that the pulmonologist can safely administer and perform the bronchoscopy, as at most places the procedure is done without the presence of anaesthesiologists. Hence, we used lower dose of midazolam in our study. However, the results of our study demonstrate that, at 0.02 mg/kg, the drug does not provide suitable patient satisfaction or bronchoscopy conditions though it did not produce any serious adverse effects.

Studies have highlighted the utility of dexmedetomidine IV (1 *µ*g/kg bolus over 10 min) as a safe and effective agent in complementing topicalisation of airway for awake fiberoptic intubation of trachea by anaesthesiologists [17–19]. Though an infusion of dexmedetomidine is usually considered during attempts at intubation, we decided to use only bolus dexmedetomidine for our study due to several reasons: (a) the procedure of flexible bronchoscopy is much less stimulating to patients than the procedure of tracheal intubation; (b) it would be convenient for the bronchoscopist to give one bolus dose and proceed to bronchoscopy; and (c) it may limit the adverse effects. As our study results demonstrate, a single bolus of dexmedetomidine at 1 *µ*g/kg over 10 min prior to bronchoscopy was sufficient to provide safe but effective sedation. A study that included infusion of dexmedetomidine at 0.5 *µ*g/kg/h after initial bolus showed frequent instances of hypoxaemia (14%, oxygen saturation SpO_2_ < 90% for >30 s) despite nasal oxygen supplementation throughout the procedure of bronchoscopy with 6% incidence of hypotension (SBP < 90 mmHg or mean blood pressure < 60 mmHg) [20]. In another study where anaesthesiologists monitored and managed the patients using combination of propofol and dexmedetomidine for sedation during bronchoscopy, the patients were first premedicated with 0.03 mg/kg IV midazolam for topicalisation of airway and then propofol 0.5 mg/kg bolus was administered followed by dexmedetomidine bolus 0.05 mL/kg of 4 *µ*g/kg preparation, which was further titrated as infusion at 0.4–2 *µ*g/kg/h during the procedure. All patients received oxygen supplementation via nasal cannula. The study found one out of 35 patients having oxygen desaturation during the procedure requiring verbal/tactile stimulation to maintain the airway. However, patients appeared to be more sedated than conscious sedation during the procedure (MOAA/S scale of 4) as 27/35 could respond only to prodding and shaking and 5/35 patients were responsive only after their name was called out loudly or repeatedly or both (MOAA/S scale of 3) five minutes after the bronchoscopy. Both of these features are undesirable when an anaesthesiologist is not present for monitoring the patient [14].

Numerical rating score for pain and distress revealed unacceptable degree of pain and distress in 11 patients who received midazolam as the study drug while one patient had unacceptable pain response and two patients had unacceptable distress levels with dexmedetomidine. Further, moderate degree of distress persisted in eight patients receiving midazolam as study drug even 10 min after the bronchoscopy was over. The majority of patients receiving dexmedetomidine had adequate sedation (25/27) during the procedure while 16 patients receiving midazolam had adequate sedation. However, four patients receiving dexmedetomidine and one patient receiving midazolam (with three top up rescue doses) had unacceptably deeper level of sedation even after 10 minutes following the procedure. All these patients were followed up and found to have recovered to awake state in the following 30-minute period without any side effects.

Haemodynamic parameters were assessed during the procedure. All but one patient had <30% deviation from baseline systolic blood pressure or heart rate in either group. One patient in the dexmedetomidine group had significant reduction in the systolic blood pressure that responded to a single crystalloid bolus of 250 mL.

A study that used combination of propofol with dexmedetomidine demonstrated that there were lower instances of oxygen desaturation with this combination as compared to propofol remifentanil combination [14]. Several studies that have used higher doses of midazolam (>0.02 mg/kg) have documented risk of oxygen desaturation during the procedure despite prophylactic oxygen administration to all patients [11, 12, 21, 22]. In one study, midazolam in a dose of 0.07–0.1 mg/kg IV administered two minutes prior to bronchoscopy resulted in episodes of desaturation to 85% despite O_2_ therapy, requiring administration of higher O_2_ concentrations as well as requirement for flumazenil to reverse the effects of midazolam in one patient [11]. None of the patients in our study had any episode of desaturation (SpO_2_ < 93%). Patient's own evaluation would probably be the best way to judge the comfort levels. Therefore, all patients were asked to give their feedback on the quality of sedation and distress experienced in a postoperative questionnaire that was asked 24 h following the procedure (to ensure that the patient was completely out of the effects of the study drugs). This also confirmed the study findings that the majority of the patients who received dexmedetomidine as the study drug were satisfied with the quality of sedation and the majority of patients experienced nil or minimal discomfort during the procedure. These findings show that a single bolus of dexmedetomidine (1 *µ*g/kg over 10 min) administered just prior to bronchoscopy provides acceptable sedation without any serious side effects.

### 5.1. Drawbacks of Our Study

One of the important drawbacks of our study probably is the timing of IV midazolam. Several studies have administered IV midazolam bolus about three minutes prior to bronchoscopy [12, 23]. It is not clear if they performed airway topicalisation prior to or following administration of midazolam. Midazolam was administered following airway topicalisation in one study [21]. During flexible bronchoscopy, maximal stimulation and patient discomfort can occur when the bronchoscope is in contact with vocal cords, trachea, carina, or bronchi as these are the most sensitive areas for foreign body sensation and are rich in irritant receptors. With our previous experience of fiberoptic bronchoscopy we felt the fiberscope will be passing through the vocal cords into the trachea approximately 2-3 min after the application of lignocaine jelly to nostril as this includes (entry of fiberscope into the nostril, its passage to nasopharynx, lignocaine spray to vocal cords and throat, and a waiting period of one minute prior to the entry of fiberscope through the vocal cords). Therefore, we injected IV midazolam bolus 2 min prior to the start of bronchoscopy anticipating that approximately 5 min would elapse by the time the fiberscope is in contact with vocal cords from the time of midazolam administration. However, the literature evidence shows that the peak effect of IV midazolam is around 5–10 min from the time of its administration [5]. Our aim was to compare the dose of midazolam recommended for sedation (0.01–0.1 mg/kg), though the dose used in our study was in the lower part of that range [5]. Further, the dose of midazolam might not be equipotent to that of dexmedetomidine. Another important limitation of the study was inability to ensure complete blinding of the individual performing bronchoscopy to group allocation as the haemodynamic profile of patients receiving dexmedetomidine was different from that of midazolam.

## 6. Conclusions

During flexible fiberoptic bronchoscopy under topical airway anaesthesia, IV dexmedetomidine (1 *µ*g/kg administered 10 min prior to bronchoscopy) provides superior patient comfort and tolerance as compared to IV midazolam (0.02 mg/kg). However, monitoring of the patients in the immediate postprocedure period is essential when dexmedetomidine is used in the present dose.

## Figures and Tables

**Table 1:** Composite score during bronchoscopy (5–25).

Parameter	1	2	3	4	5
Sedation	Awake to voice >10 s	Light sedation, briefly awakens to voice	Moderate sedation, no eye contact	Deep sedation, response only to physical stimulation	Unarousable, no response to physical stimulation

Calmness	Alert and calm	Anxious, not aggressive	Frequent nonpurposeful movement	Pulls or removes bronchoscope; aggressive	Combative, violent

Respiratory response	Spontaneous respiration No coughing	Occasional cough	Coughing regularly	Frequent coughing or chocking	Vigorous cough preventing bronchoscopy

Physical movement	No movement	Occasional slight movement	Frequent slight movements	Vigorous movement limited to the extremities	Vigorous movement including torso and head

Facial tension	Facial muscle tone normal	Mild muscle tension evident	Tension evident in all facial muscles	Grimacing	Grimacing and crying

**Table 2:** Patients' characteristics and bronchoscopy data.

Characteristics and data	Group M Mean ± SD	Group D Mean ± SD	*p* value
Age (years)	52.52 ± 15.05	49.70 ± 13.85	0.478^#^
Gender (M/F)	24/3	20/7	0.161^*∗*^
Height (cm)	164.89 ± 5.78	160.00 ± 5.04	0.002^#^
Weight (kg)	56.07 ± 10.84	53.26 ± 10.10	0.328^#^
BMI (kg/m^2^)	20.42 ± 3.46	20.77 ± 3.40	0.713^#^
Procedures	Diagnostic (27)	Diagnostic (27)	1
Duration of bronchoscopy	8.19 ± 2.53	8.93 ± 2.96	0.327^#^

^*∗*^Chi-square test.

^#^Independent *t*-test.

**Table 3:** Comparison of composite score (total score).

Total score	Group M	Group D
	(*n* = 27)	(*n* = 27)
5	0	1
6–10	4	19
11–15	11	6
16–20	12	1
Mean ± SD	14.48 ± 3.65	9.41 ± 3.13

*p* < 0.001 (Mann-Whitney *U* test).

**Table 4:** Split-up of components of the composite score.

Component of composite score	Group M		Group D	
	(*n* = 27)		(*n* = 27)	
	Number	%	Number	%
Sedation				
1	23	85.2	22	18.5
2	4	14.8	4	70.4
3	0	0	0	11.1
4	0	0	1	0.0
5	0	0	0	0.0
Calmness				
1	1	3.7	8	29.6
2	8	29.7	13	48.2
3	11	40.7	4	14.8
4	7	25.9	2	7.4
5	0	0	0	0
Respiratory response				
1	1	3.7	5	18.5
2	4	14.8	19	70.4
3	15	55.6	3	11.1
4	6	22.2	0	0.0
5	1	3.7	0	0.0
Physical movement				
1	1	3.7	6	22.2
2	5	18.5	15	55.6
3	4	14.8	5	18.5
4	9	33.3	0	0.0
5	8	29.7	1	3.7
Facial tension				
1	0	0.0	5	18.5
2	4	14.8	17	63.0
3	8	29.6	3	11.1
4	7	25.9	0	0.0
5	8	29.6	2	7.4

**Table 5:** Numerical rating scale for pain and distress.

	Prior to	During			10 min after bronchoscopy		
NRS pain		0–3	4–7	8–10	0–3	4–7	8–10
Group M	0	8	8	11	26	1	0
Group D	0	22	4	1	27	0	0

		**p value < 0.001** (*Chi-square test*; *statistically significant*)			**p value** = **1** (*Fisher's exact test*)		

NRS distress		0–3	4–7	8–10	0–3	4–7	8–10
Group M	0	4	12	11	19	8	0
Group D	0	19	6	2	26	1	0

		**p value < 0.001** (*Chi-square test*; *statistically significant*)			**p value** = **0.024** (*Fisher's exact test*; *statistically significant*)		

**Table 6:** Various other study parameters.

Ramsay sedation score			
Time in relation to bronchoscopy	RSS	Group M	Group D
		(*n* = 27)	(*n* = 27)
Prior to	2	25	17
	3	2	7
	4	0	3

	**p value** = **0.024** (*Fisher's exact test*; *statistically significant*)		

During	1	11	2
	2	15	24
	3	1	1

	**p value** = **0.009** (*Fisher's exact test*; *statistically significant*)		

10 min after	2	2	0
	3	24	23
	4	1	3
	5	0	1

	**p value** = **0.356** (*Fisher's exact test*)		

Ease of bronchoscopy			
Easy		6	15
Slightly difficult		11	10
Very difficult		10	2

		**p value** = **0.010** (*Chi-square test*; *statistically significant*)	

*Patient's opinion 24 h after the bronchoscopy*			
Quality of sedation			
Excellent		0	5
Good		7	16
Fair		16	6
Poor		4	0
Amount of discomfort			
Nil		2	7
Mild		10	16
Moderate		10	4
Severe		5	0
Number of rescue midazolam boluses required			
1		8	2
2		1	0
3		1	0

## Key Message

Dexmedetomidine appears to be the answer for the search for a rapid acting and short duration medication that improves patient tolerance to flexible bronchoscopy without jeopardising patient safety.
